# Congruence and discrepancy in Asian American women's perception and stress appraisal of gendered racial microaggressions: Relationships with depressive symptoms and internalized racism

**DOI:** 10.3389/fpubh.2022.954897

**Published:** 2022-10-25

**Authors:** Brian TaeHyuk Keum, Michele J. Wong

**Affiliations:** Department of Social Welfare, UCLA Luskin School of Public Affairs, University of California, Los Angeles, Los Angeles, CA, United States

**Keywords:** Asian American women, gendered racism, internalized racism, depressive symptoms, response surface analysis, gendered racial microaggressions

## Abstract

Prior research demonstrates significant links between discrimination and mental health by assessing either encounters with or stress appraisal of discrimination. However, research has yet to examine the dynamic interplay between frequency and stress appraisal (e.g., high frequency-low stress appraisal) and their linkage to depressive symptoms. Using a sample of 309 Asian American women (*M*_*age*_ = 22.81, *SD* = 0.26), we used a polynomial regression and response surface analysis to model the congruence and discrepancy between frequency and stress appraisal of gendered racial microaggressions experienced by Asian American women and how they are related to depressive symptoms and internalized racism. The dynamics between frequency and stress in relation to depressive symptoms were further probed at low, mean, and high levels of internalized racism. Greater congruence between frequency and stress was significantly associated with depressive symptoms (medium to large effect) and internalized racism (small effect). A discrepancy between higher frequency and lower stress was significantly associated with greater internalized racism. Further, when looking across levels of internalized racism, greater congruence between frequency and stress was significantly associated with greater depressive symptoms at low and mean levels of internalized racism but not at high levels. Gendered racial microaggressions are associated with adverse mental health outcomes among Asian American women, contributing to greater depressive symptoms and internalized racism. Further consideration should be given toward how internalized racism shapes differences in the perceptions and stress appraisal of gendered racial microaggressions, and subsequent mental health outcomes among Asian American women.

## Introduction

The 2021 Atlanta spa shootings and the ongoing attacks and murders of Asian American women [AAW; (1)] are hate crimes that must be contextualized within the dominant sexist and racist narratives about AAW in the United States. Based on the intersectionality framework, which views multiple social identities (e.g., race, gender, class, etc.) within the context of interlocking systems of oppression that uniquely effect marginalized groups' experiences (2) and the concept of gendered racism, which examines experiences of oppression at the intersection of gender and race (3), gendered racial microaggressions (GRM) highlight the oppression that Asian American women experience within the context of the long standing gendered racial stereotypes of AAW as submissive, fetishized, invisible, and domesticated women (4). In particular, these stereotypes have historically rendered AAW as subservient objects of sexual fetishization (“yellow fever”) among White men (5, 6) and have placed them at great risk for potential sexual and dating violence, as well as physical assault. Given the pernicious and chronic manifestation of microaggressions in the daily lives of racial minority individuals, emerging literature suggests that GRM is linked to a host of mental health issues including depressive symptoms and suicide ideation among AAW (4, 7).

Suicide remains one of the leading causes of death among Asian American women (AAW), particularly AAW in their late adolescence and emerging adulthood [ages 15–24; (8)]. As the alarming statistics on suicide deaths among AAW continue to persist, it is imperative to examine the dynamics between GRM and depressive symptoms, a major risk factor related to suicide. One major question is how the frequency, or perceived number of GRM and stress appraisal of GRM events may play a role in developing depressive symptoms. For some AAW, they may perceive few instances of GRM but appraise them to be extremely stressful when they do occur. On the other hand, some AAW may perceive a lot of GRM, but how much stress is experienced may be complex and subdued, especially if they harbor high levels of internalized racism that pushes them to survive through these oppressive dynamics by appropriating toward the oppressors' expectations and stereotypes (9, 10). Thus, the interplay between the frequency or perceived number of GRM and the level of stress appraised merits examination of their linkage to depressive symptoms and internalized racism. This dynamic is important to examine as it can provide a nuanced understanding of how factors such as internalized racism can contextualize the varied impacts of GRM on depressive symptoms among AAW. Thus, the aim of this study was to examine the congruence and discrepancy between frequency and stress appraisal of GRM experienced by AAW and how they are related to depressive symptoms and internalized racism.

### Frequency and stress appraisal of gendered racial microaggressions

A growing body of evidence links the experience of GRM, subtle everyday instances of discrimination that denigrate individuals based on their intersecting gender and racial identities, with negative mental health outcomes such as depressive symptoms (4, 7, 11, 12). Mukkamala and Suyemoto (13) used a multimethod qualitative approach to capture the overlapping oppressions that AAW experience, highlighting themes of being seen as exotic, not a leader, submissive and passive, cute and small, invisible, and as a service worker. In efforts to advance quantitative research on the unique stressors impacting the mental health of AAW, Keum et al. (4) operationalized GRM among AAW into four key domains: (a) Ascribed Submissiveness; (b) Assumption of Universal Appearance; (c) Asian Fetishism and; (d) Media Invalidation, finding GRM to contribute unique risk to depressive symptoms above and beyond general experiences of sexism and racial microaggressions among a sample of AAW. These findings align with prior work that draws from the stress and coping model, conceptualizing racial discrimination to adversely impact health when the perceived stressors from discrimination exceeds available personal and social coping resources (14–16).

Research that draws from the stress and coping model to examine the link between racial discrimination and health among Asian Americans often assesses the presence or absence of discriminatory events, which assumes a greater frequency of reported discrimination leads to greater stress and worse health (17, 18). The Everyday Discrimination Scale (EDS; 14), a measure that is often employed in public health research, uses a nine-item scale to capture chronic and routine instances of unfair treatment (e.g., treated with less respect, called names or insulted) (19). Despite having strong psychometric properties (20, 21), the EDS remains limited in its ability to appraise the stressfulness of the chronic instances of unfair treatment (22). Alternatively, measures like the General Ethnic Discrimination Scale [GED; (18)] examine dimensions of chronicity and stress by asking respondents how often they encountered racial discrimination in the past year and over their lifetime, as well as how stressful the encounter was (18). While the two ratings allow for the evaluation of the frequency and stress of a particular racial discrimination event, studies have yet to examine the concurrent interplay between the two dimensions. According to the stress response framework of racism's impact, as a racial minority individual encounters a certain level of racism in their daily lives, how much stress is derived from these experiences on an ongoing basis would be important to understand regarding implications of psychopathology. Thus, a more dynamic understanding of encounters with GRM and their perceived stressfulness is needed to illuminate differences in the stress appraisal process for Asian American women exposed to GRM; specifically, why some AAW may find GRM experiences such as AAW fetishization to be very stressful, whereas other AAW may experience very little stress.

### Congruence and discrepancy in perception and stress appraisal of gendered racial microaggressions

For AAW who experience GRM, there are several factors that may vary their perceptions of GRM events and differentiate their stress appraisal. In particular, research has indicated that factors such as nativity status, generational status, and years in the U.S. can shape how individuals perceive discrimination. For example, in a study that examined experiences of perceived discrimination and wellbeing among a sample of U.S.-raised Asian students (born in the U.S., spent more than half their age in the U.S., or moved to the U.S. before age 12) and non-U.S.-raised Asian students (Asian students born or raised outside of the U.S.), findings indicate that U.S.-raised students reported greater degrees of acculturation and significantly higher scores on perceived discrimination compared to non-U.S. raised students (23). The acculturation process for many Asian Americans provide a way to cope with their perceived differences as they navigate White cultural norms that are often associated with being American (23, 24). Unfortunately, much of this process involves exposure to various forms of U.S. racism and discrimination embedded within mainstream American culture that can negatively impact the mental health of more acculturated Asian Americans who are accustomed to identifying and understanding the implications of their encounters with racism (25, 26). Indeed, research indicates that U.S.-raised Asian students experiencing racial discrimination report more depressive symptoms, less life satisfaction, and lower self-esteem mediated through denial of their American identity (27). Hence, AAW raised in the U.S. may be more attuned to experiences of GRM as discriminatory, such that increases in exposure to GRM are linked to a congruent increase in their stress response, which can lead to worse mental health.

However, few studies have examined factors that may shape discrepant experiences between discrimination and stress appraisal. Specifically, some AAW may encounter a lot of GRM, yet experience very little or no stress in response to these incidents. Conversely, for some AAW, these same encounters with GRM may be very stressful for them. Thus, AAW's stress response depends on how they perceive GRM, which can vary depending on their level of awareness of GRM as well as their ability to cope. There is some research to suggest that color-blind racial attitudes, the belief that race or racism should not matter, can shape how Asian Americans perceive racial dynamics and the existence of racism in the U.S. (28). For instance, first and 1.5 generation Asian Americans have been found to have limited awareness regarding instances of blatant racism (29). These findings are consistent with research among non-U.S.-raised who report higher scores on racial color blindness (23). Further, there is evidence that non-U.S.-raised Asian individuals who perceive foreigner objectification (e.g., perpetual foreigner stereotype) do not experience the same associations with depressive symptoms, low life satisfaction, and low self-esteem as their counterparts (27). Some scholars have suggested that color-blind racial attitudes among Asian Americans that were not raised in the U.S. may relate to coming from a more homogenous culture where racial/ethnic identity holds less relevance, and they are yet to be socialized or become critically conscious of the racial dynamics in the U.S. (29, 30). Further, they may experience a process of enculturation where they work to maintain the values and norms of their culture of origin as they navigate American culture (31). However, color-blind racial attitudes may still be common among Asian Americans who are second generation or beyond, suggesting more complex processes that may be tied to internalized racism among Asian Americans who are deeply embedded in mainstream American culture and U.S. racism (29). Despite the importance of examining the role of internalized racism in influencing the mental health of Asian Americans, few studies have explored the significance of internalized racism among AAW. Thus, this study seeks to elucidate the role of internalized racism among AAW, particularly how it may influence discrepancies experienced between the perceived number of GRM encounters and stress appraisal.

### Internalized racism and gendered racial microaggressions

A growing body of research among Asian Americans demonstrates a link between internalized racial oppression and adverse mental health outcomes, such as increased depressive symptoms and psychological distress (9, 32, 33). Briefly, internalized racial oppression can be understood as the conscious or unconscious adoption of racist stereotypes, ideologies, and values perpetuated by the White dominant society about racial/ethnic minorities, and can manifest through self-hatred, low self-worth, and the acceptance of negative or positive stereotypes of one's self or racial group (32–34). Scholars have argued that internalized racial oppression is better conceptualized and termed appropriated racial oppression, as it shifts the attention away from emphasizing internal factors to focusing on oppression as systemic (9, 10). As such, individuals are not limited to passively accepting the negative messaging associated with one's racial group but may use various “tools of oppression” to adapt and respond to normative Whiteness (e.g., assimilation, code-switching) (10, 33).

Accordingly, AAW who encounter GRM and experience internalized racial oppression may engage in the stress appraisal process in different ways. Internalized racial oppression among Asian Americans have been operationalized by Choi et al. (32) to include three general and gendered dimensions that include: (a) self-negativity, (b) weakness stereotype, and (c) appearance bias. Self-negativity may be especially detrimental for Asian Americans that express negative attitudes, low collective self-esteem, and an overall devaluation of one's own Asian American identity. Indeed, there is research to suggest a direct link between internalized racial oppression and depressive symptoms (9, 35). Further, recent research shows high levels of internalized racial inferiority exacerbate the link between racial/ethnic discrimination and psychological distress among Asian American adults (36). Hence, for some AAW, internalized racial oppression may reinforce their experience of self-negativity, particularly those who endorse negative messaging related to GRM, which may exacerbate depressive symptoms. Alternatively, there are AAW who may find ways to downplay, deny, and even justify their experiences of GRM, endorsing assumptions of their submissiveness and exoticization, and may not view GRM as discriminatory (33). A recent review of internalized racism and health among racial/ethnic minorities indicates that internalized racism can manifest in complex ways that can contribute to worse health while also serving as a self-protective strategy (34). Thus, Asian American women who appropriate negative messaging from GRM may choose to “play along” with the oppressive values perpetuated by White dominant society as a survival strategy to avoid feeling more stressed or down about themselves. Therefore, the perception and stress appraisal of GRM events may be affected by varying levels of internalized racial oppression among AAW, which may have implications for the development of mental health issues such as depressive symptoms.

### The present study

Our review of the literature suggests that there may be congruence or discrepancy between the frequency, or perceived number of GRM AAW encounter, and the intensity of stress appraised from these encounters. Examining this process is important as it can provide greater nuance into the mechanism of how GRM may impact AAW's mental health. As reviewed, the dynamic between how one encounters discrimination and subsequently experiences stress can be influenced by many social and cognitive factors, such as internalized racism. To address this gap in the literature, we examined whether the degree of congruence and discrepancy between frequency and stress appraisal of GRM among AAW would be associated with their depressive symptoms and internalized racism. We defined congruence and discrepancy as agreement and disagreement between (a) the frequency or perceived number of GRM encountered, and (b) the level of stress experienced from GRM. We employed a polynomial regression and response surface analysis [PRRSA; (37)] to test our hypotheses. PRRSA allows simultaneous modeling of (x) and (y) predictors scored on the same items (in this case, the GRM items rated for both frequency and stress appraisal) with the outcome variable (z; in this case, depressive symptoms and internalized racism), which overcomes the limitations of difference scores. PRRSA has been commonly used to test the congruence and discrepancy of social psychological constructs (38). The following were our hypotheses:

#### Hypothesis 1

We first examined how the congruence and discrepancy between GRM frequency and stress appraisal are related to depressive symptoms. We hypothesized that greater congruence (e.g., more GRM encountered being congruent with more stress experienced) would be significantly related to higher depressive symptoms. In other words, AAW who encounter and experience greater levels of GRM and its associated stress would report significantly higher depressive symptoms.

For discrepancy, we examined two trends: (a) AAW who report low GRM frequency but high stress, and (b) AAW who report high GRM frequency but low stress. We hypothesized that AAW would be more likely to report significant depressive symptoms with the former discrepancy than the latter given that the stress stemming from GRM would be a likely agent that impacts their mental health. Even if AAW encounter infrequent GRM, if they experience a high level of stress, the impact on their mental health may be significant. If AAW encounter frequent GRM but reports little stress, there may be less impact on their mental health.

#### Hypothesis 2

Regarding internalized racism as the outcome variable, we examined whether the discrepancy between GRM frequency and stress appraisal is related to internalized racism. We hypothesized that the high frequency-low stress discrepancy would be significantly associated with higher internalized racism than the low frequency-high stress situation. Based on our review, for AAW with a high level of internalized racism, they may be less aware and attuned to recognizing the harmful costs of GRM [e.g., (33)]. Alternatively, AAW may have adapted to forms of normative Whiteness, where they find ways to actively engage with the negative messaging associated with GRM and find a way to use it to their advantage, thereby limiting GRM from contributing to more stress (10, 33).

For congruence, our analysis was exploratory. On one hand, we anticipated that lower congruence (e.g., less GRM encountered being associated with less stress experienced) may be significantly related to higher internalized racism. Based on the evidence and theory that those with high internalized racism may be less attuned to recognition of GRM and may even deny the harmful costs of GRM, or that AAW has found a way to actively engage in the negative messaging associated with GRM to use it to their advantage to minimize the stress they may otherwise experience, AAW who perceive lower levels of GRM and its associated stress may be associated with higher internalized racism. On the other hand, it is also possible that greater congruence (e.g., more perceived GRM being congruent with more stress experienced) would be significantly related to higher internalized racism as more experiences with GRM may push AAW to internalize negative messages about their Asian identity.

#### Hypothesis 3

Subsequently, we examined how the congruence and discrepancy between GRM frequency and stress appraisal are related to depressive symptoms across low (−1 SD), mean, and high (+1 SD) levels of internalized racism. At low and mean internalized racism levels, we hypothesized that greater congruence would be significantly related to higher depressive symptoms but not for those with high internalized racism. For discrepancy, we hypothesized that low frequency-high stress discrepancy would be significantly related to depressive symptoms (vs. high frequency-low stress) at low and mean levels. At a high level of internalized racism, we hypothesized that neither congruence nor discrepancy may be significantly associated with depressive symptoms.

## Methods

### Participants and procedure

The current sample was a subset of data from a previous study (Authors anonymous) that was conducted in compliance with the Institutional Review Board. The inclusion criteria were: (a) 18 years old or older and (b) currently enrolled in an APA-accredited doctoral program in counseling psychology (incoming students or alumni were ineligible). Data was collected *via* an online survey consisting of informed consent, study variable measures, and demographic items hosted by Qualtrics. The survey was advertised through multiple online communication platforms such as listservs, discussion forums, and social network sites (e.g., Facebook) pertaining to Asian American women.

Participants were 309 adult Asian American women ranging from 18 to 68 years old (*M* = 22.81, *SD* = 6.26). The majority (99%) identified as a cisgender woman, and the remaining participant identified as Genderfluid. About 83% identified as heterosexual, 7% bisexual, 4% uncertain or questioning, 2% Queer, 2% Asexual, 1% Lesbian, and 1% Other. The sample was diverse in ethnicity: Chinese (29%), Korean (16%), Indian (10%), Vietnamese (7%), Taiwanese (7%), Filipina (6%), Japanese (2%), and 17% Multiracial. The remaining 6% identified as Native Hawaiian/Pacific Islander, Cambodian, Thai, Hmong, Laotian, Bangladeshi, and Indonesian. Majority (74%) identified as second generation and beyond, while the remaining participants (36%) identified as 1st or 1.5 generation. In terms of education, majority (69%) had some college or earned a college degree, 20% earned a graduate (e.g., Master's) or professional degree (e.g., MD), and 11% had high school diploma. The majority of the sample (51%) identified as middle class, followed by upper-middle class (27%), working class (16%), lower class (4%), and upper class (2%).

### Measures

#### Gendered racial microaggressions stress

The Gendered Racial Microaggressions Scale for Asian American Women [GRMSAAW; (4)] is a 22-item scale that assesses the behavioral, verbal, and environmental manifestations of GRM experienced by AAW in the United States. The four subscales are: (a) Ascribed Submissiveness; microaggressions rooted in submissive stereotypes and assumptions of AAW, (b) Assumption of Universal Appearance; stereotypes and assumptions that minimize and confine all AAW's body image and appearance attributes to certain “Asianized” standards, (c) Asian Fetishism; sexual objectification and fetish (e.g., yellow fever), and (d) Media Invalidation; underrepresentation and negative stereotypical portrayals in the media. The general factor (total score) represented unique shared variance across all items. We used the frequency (0 = *Never*, 1 = *Rarely*, 2 = *Sometimes*, 3 = *Often*, 4 = *Very frequently*, 5 = *Always*) and stress appraisal (0 = *not at all stressful*, 1 = *Slightly stressful*, 3 = *Moderately stressful*, 4 = *Very stressful*, 5 = *Extremely stressful*) total scale scores (GRMS). Higher frequency scores indicate greater amounts of GRM experienced and higher stress scores greater stress experienced from GRM. Sample items include “Others express sexual interest in me because of my Asian appearance,” and “I rarely see AAW in the media.” Keum et al. (4) reported good internal consistency with Cronbach's alphas ranging from 0.86 to 0.94 for stress appraisal. Construct validity was supported by associations with racial microaggressions, sexism, depression, and internalized racism scores (4). The total score internal consistency for the current study was 0.91 and 0.91 for frequency and stress.

#### Depressive symptoms

Patient Health Questionnaire-9 [PHQ-9; (39)] is a 9-item depression scale that establishes provisional depressive disorder diagnoses and depressive symptom severity. Participants respond on a 4-point Likert-type scale (0 = *not at all* to 3 = *nearly every day*). Scores range from 0 to 27 with higher scores indicating more severe depression. The PHQ-9 exhibits convergent validity and sensitivity to change; scores on the PHQ-9 were correlated with the Symptom Checklist-20 and changes in PHQ-9 scores were similar or greater than change in SCL-20 (39). Validity and measurement invariance of PHQ-9 with Asian American college students has been confirmed (40). Internal consistency for the total scale score in our sample was 0.91.

#### Internalized racism

The Internalized Racism in Asian American Scale [(32); IRAAS] is a 14 item 3-factor scale that measures the degree to which Asian Americans internalized hostile attitudes and negative messages regarding their racial identity. The three subscales are: (a) Self-negativity; global devaluation and negative attitudes directed toward one's own Asian American identity, (b) Weakness Stereotypes; internalized beliefs in negative stereotypes of deficit or weakness inherent to being Asian American, and (c) Appearance Bias; endorsement of Eurocentric standards of attractiveness and downward comparisons of Asian Americans. The total scale score was used in the current study. Responses are based on a 6-point Likert-type scale (1 = *strongly disagree* to 6 = *strongly agree*), with higher scores indicating greater internalized racism. Positively worded items were reverse-scored. A sample item is “I sometimes wish I weren't Asian.” Convergent validity was assessed by correlating scores with Collective Self-Esteem Scale and authors indicated IRAAS was valid. Predictive validity was also demonstrated; high levels of internalized racism were significantly predictive of depressive symptoms. The internal consistency for the total scale score was 0.88.

### Data analysis

**Figure 1:**
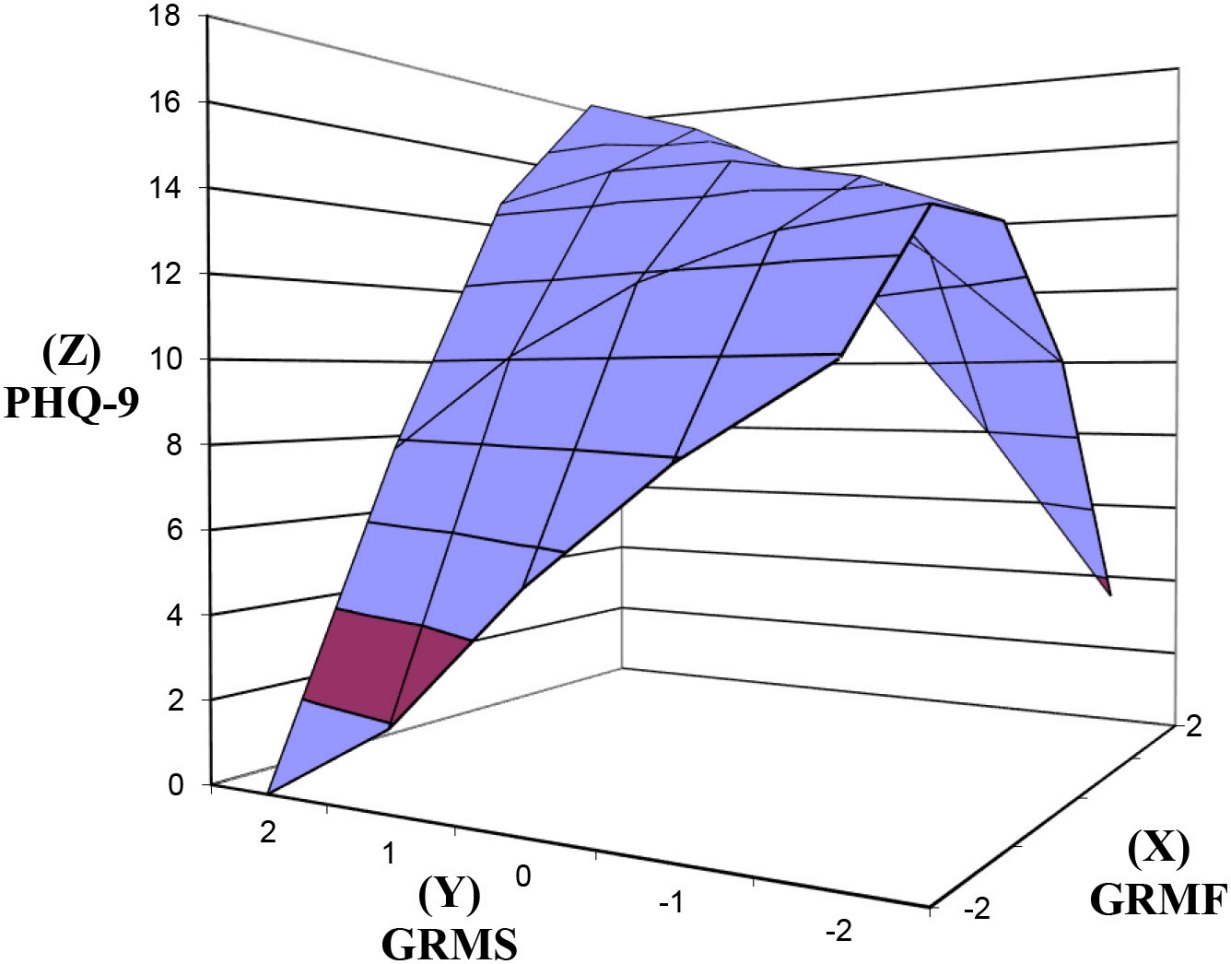
Response surfaces predicting PHQ-9 as a function of congruence and discrepancy between GRMF and GRMS. PHQ-9, Patient Health Questionnaire-9; GRMF, Gendered Racial Microaggressions Frequency; GRMS, Gendered Racial Microaggressions Stress; Line of congruence (X = Y; back to front); Line of discrepancy (X = –Y; left to right); point of lowest congruence (−2, −2; front corner); point of highest congruence (2, 2; back corner); congruence increases from front to back corner; right most corner (2, −2) = discrepancy between high GRMF-low GRMS; left most corner (−2, 2) = discrepancy between low GRMF and high GRMS.

We used polynomial regression and response surface analysis [PRRSA; (37)] to model the three-dimensional representation (e.g., Figure 1) of the congruence between GRM frequency (GRMF; X) and stress appraisal (GRMS; Y). PRRSA was the method of choice because it addresses the limitations of difference scores (i.e., low reliability and loss of unique outcome interpretations when the same item is rated across time or by the same participant) by retaining and modeling the independent effects of the two predictors simultaneously with the outcome variable [Z; (37)].

Following Shanock et al. (37), we created three new variables for the X and Y predictor variables: (a) square of the centered GRMF, (b) cross-product of the centered GRMF and GRMS, and (c) square of the centered GRMS. The three new variables, along with the X (GRMF) and Y (GRMS) predictor variables were entered into a polynomial regression model predicting the outcome variables (Z; PHQ-9, IRAAS) to obtain regression (gamma) coefficients and standard errors, which were used to generate the response surfaces (e.g., Figure 1) and coefficients for the slopes and curvatures above the lines of congruence and discrepancy. Social class and generational status were also entered into the model as covariates. The spreadsheet provided by Shanock et al. (37) was used to generate the coefficients and the surface figures.

The equation for the model was:

PHQ9/IRAAS = *b*_0_ + *b*_1_ *GRMF* + *b*_2_ *GRMS* + *b*_3_
*GRMF*^2^ + *b*_4_ GRMF^*^GRMF + *b*_5_ *GRMS*^2^ + *e*.

We tested our hypotheses by examining the significance of congruence and discrepancy (across lines of congruence and discrepancy) between the X and Y predictor variables in relation to the outcome variables (Z). The degree of congruence is indicated on the line of congruence that extends from congruently low values (−2, −2; i.e., front corner in Figure 1) to congruently high values (2, 2; i.e., back corner in Figure 1). A significant positive slope along the line of congruence (x = y) would confirm the hypothesis that congruently higher X (GRMF) and Y (GRMS) variable scores are significantly associated with higher Z (e.g., PHQ), whereas a negative slope would indicate a decrease in Z.

The degree of discrepancy is indicated on the line of discrepancy that extends from a point of lowest X and highest Y values (−2, 2; i.e., left corner in Figure 1) to a point of highest X and lowest Y values (2, −2; i.e., right corner in Figure 1). Along the line of discrepancy (x = –y), a significant negative slope would confirm the hypothesis that Z (e.g., PHQ-9) is higher when Y (GRMS) is high and X (GRMF) is low, rather than a discrepancy due to low GRMS (Y) and low GRMF (X). On the other hand, a positive slope would indicate that the reverse is evident.

Although linear associations between the variables were the focus of the study, tests of the curvatures of the response surfaces are provided as part of the PRRSA. A significant curvature along either the line of congruence or the line of discrepancy indicates a non-linear response surface. A negative curvature indicates a concave surface that is downward curving while a positive curvature indicates a convex surface that is upward curving. Both curvatures can be used to infer whether outcomes increase or decrease sharply depending on congruence or discrepancy. We examined any significant curvatures in interpreting the results of the slopes.

## Results

### Preliminary analyses

Using the criteria where kurtosis and skewness between −2 and +2 suggest normal distribution (41), data were generally normally distributed except for IR (kurtosis = 3.31), suggesting that participants generally reported high levels of internalized racism. Because most variables showed conformity to the normal distribution and transforming data also carry the risk of impacting the interpretation of results, we elected to not undertake a data transformation procedure. Examination of the Q-Q plot suggested linear relationships between the independent and dependent variables. The correlation between GRMS and GRMF was 0.85, which was expected given that it is an association based on the same items. Shanock et al. (37) suggest that no multicollinearity (VIF < 5) is a requirement to conduct the RSA. The VIF value was 3.56 between GRMS and GRMF, which was within the range for evidence of no multicollinearity. Descriptive statistics and bivariate correlations of study variables are listed in Table 1.

**Table 1:** Descriptive statistics and bivariate correlations of study variables.

	**Descriptives**						**Correlations**			
**Variables**	***M***	***SD***	**Min**	**Max**	**Skewness**	**Kurtosis**	**1**	**2**	**3**	**4**
1. GRMF	2.77	0.97	0	5.00	−0.05	−0.36	–			
2. GRMS	2.81	1.13	0	5.00	−0.40	−0.49	0.85^**^	–		
3. PHQ-9	16.22	5.93	9.00	35.00	1.06	0.59	0.28^**^	0.27^**^	–	
4. IRAAS	2.43	1.01	1.00	7.00	1.38	3.31	0.11	0.03	0.25^**^	–

GRMF, Gendered racial microaggressions-frequency; GRM, Gendered racial microaggressions-stress; PHQ-9, Patient health questionnaire-9; IRAAS, Internalized racism in Asian Americans scale.

** p < 0.01.

### GRMF GRMS congruence and discrepancy in relation to PHQ-9

As seen in Figure 1, above the line of congruence (x = y), the response surface rises upward toward the top back corner of the graph [xy coordinates (2, 2)] where GRMF (X) and GRMS (Y) are both high and PHQ-9 (Z) is high. In support of hypothesis 1, the increase in PHQ-9 as GRMF GRMS congruence increases was significant (Table 2), as reflected in the significant positive slope for the response surface above the line of congruence (x = y) = 1.96, *SE* = 0.38, *t* = 5.196, *p* < 0.001. The effect size was medium to large, Cohen's *d* = 0.60. The curvature above the line of congruence (x = y) was not significant.

**Table 2:** Congruence and discrepancy slopes and curvatures of the response surfaces.

**Effect**	**Coefficient**	***SE***	***t***	***p*-value**
**DV (z)** **=** **PHQ-9**				
a1: Level of congruence predicting DV (x = y slope)	1.96	0.38	5.20	<0.001
a2: Non-linearity of congruence (x = y curvature)	−0.03	0.28	−1.07	0.284
a3: Level of discrepancy predicting DV (x = –y slope)	1.38	1.24	1.12	0.264
a4: Non-linearity of discrepancy (x = –y curvature)	−3.59	2.30	−1.56	0.120
**DV (z)** **=** **IRAAS**				
a1: Level of congruence predicting DV (x = y slope)	0.15	0.06	2.48	0.014
a2: Non-linearity of congruence (x = y curvature)	−0.07	0.05	−1.27	0.205
a3: Level of discrepancy predicting DV (x = –y slope)	0.60	0.22	2.75	0.006
a4: Non-linearity of discrepancy (x = –y curvature)	−0.54	0.40	−1.34	0.181
**DV (z)** **=** **PHQ-9 at Low IRAAS**				
a1: Level of congruence predicting DV (x = y slope)	2.01	0.84	2.39	0.021
a2: Non-linearity of congruence (x = y curvature)	−0.04	0.73	−0.06	0.953
a3: Level of discrepancy predicting DV (x = –y slope)	−2.67	3.73	−0.71	0.479
a4: Non-linearity of discrepancy (x = -y curvature)	−3.87	5.97	−0.65	0.520
**DV (z)** **=** **PHQ-9 at Mean IRAAS**				
a1: Level of congruence predicting DV (x = y slope)	1.63	0.42	3.89	<0.001
a2: Non-linearity of congruence (x = y curvature)	−0.07	0.30	−0.24	0.813
a3: Level of discrepancy predicting DV (x = –y slope)	1.24	1.40	0.89	0.374
a4: Non-linearity of discrepancy (x = –y curvature)	−4.84	2.86	−1.70	0.091
**DV (z)** **=** **PHQ-9 at High IRAAS**				
a1: Level of congruence predicting DV (x = y slope)	3.78	1.94	1.95	0.058
a2: Non-linearity of congruence (x = y curvature)	−1.80	1.29	−1.39	0.171
a3: Level of discrepancy predicting DV (x = –y slope)	5.62	5.31	1.06	0.296
a4: Non-linearity of discrepancy (x = –y curvature)	−6.16	7.49	−0.82	0.416

DV, dependent variable; PHQ-9, Patient health questionnaire-9; IRAAS, Internalized racism in Asian American scale.

Regarding the discrepancy between GRMF and GRMS, neither the slope nor the curvature was significant in predicting PHQ-9 (see Table 2). Thus, the degree of GRMF GRMS discrepancy was not significantly predictive of PHQ-9 regardless of the nature of the discrepancy (high GRMS-low GRMF or low GRMS-high GRMF). Overall, the model accounted for XX% of the variance in PHQ-9.

### GRMF GRMS congruence and discrepancy predicting IRAAS

**Figure 2:**
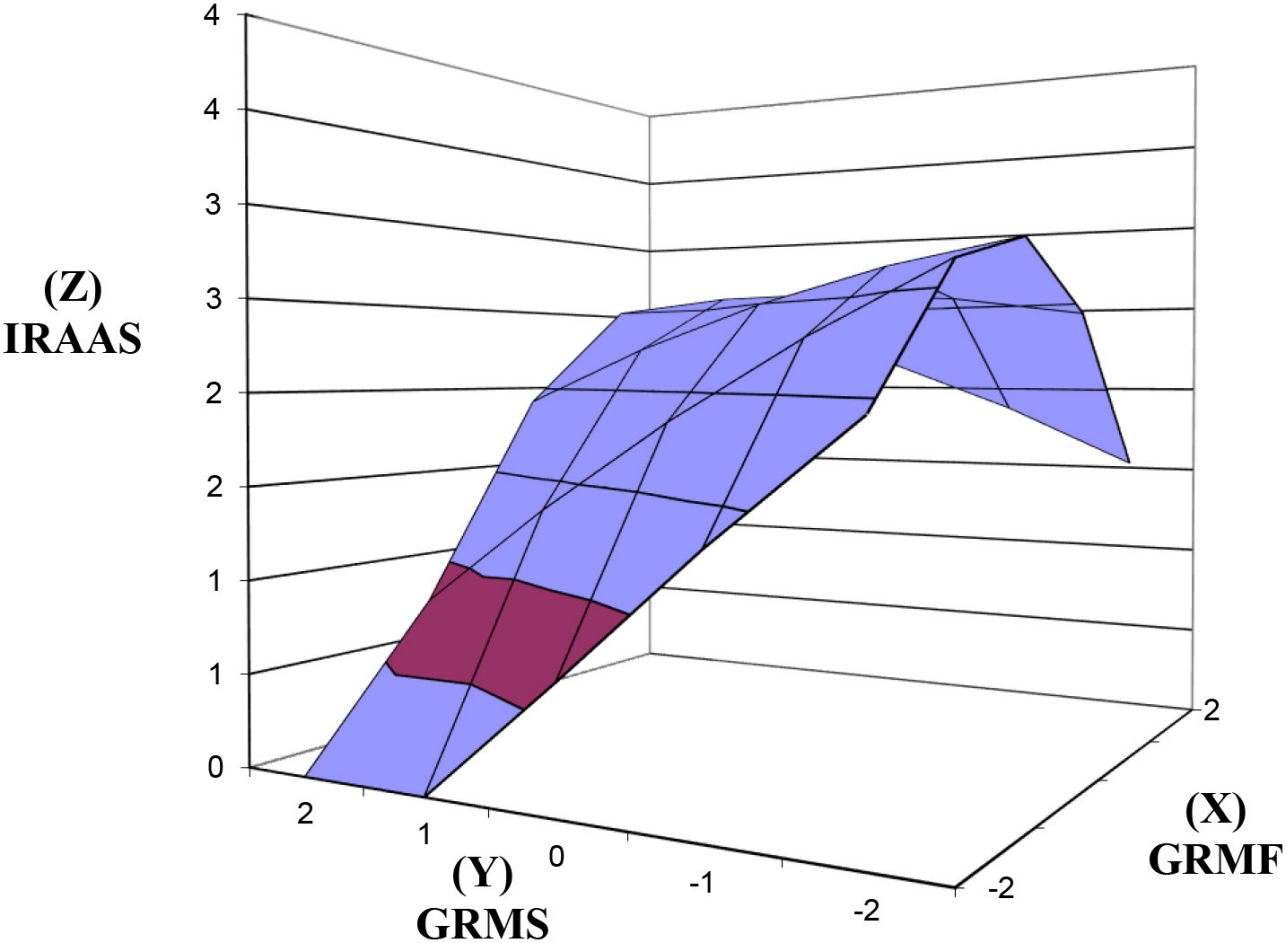
Response surfaces predicting IRAAS as a function of congruence and discrepancy between GRMF and GRMS. IRAAS, Internalized Racism in Asian American Scale; GRMF, Gendered Racial Microaggressions Frequency; GRMS, Gendered Racial Microaggressions Stress; Line of congruence (X = Y; back to front); Line of discrepancy (X = –Y; left to right); point of lowest congruence (−2, −2; front corner); point of highest congruence (2, 2; back corner); congruence increases from front to back corner; right most corner (2, −2) = discrepancy between high GRMF-low GRMS; left most corner (−2, 2) = discrepancy between low GRMF and high GRMS.

As seen in Figure 2, above the line of congruence (x = y), the response surface rises upward toward the top back corner of the graph [xy coordinates (2, 2)] where GRMF (X) and GRMS (Y) are both high and IRAAS (Z) is high. The increase in IRAAS as GRMF GRMS congruence increases was significant (Table 2), as reflected in the significant positive slope for the response surface above the line of congruence (x = y) = 0.15, *SE* = 0.06, *t* = 2.475, *p* = 0.014. The effect size was small, Cohen's *d* = 0.28. The curvature above the line of congruence (x = y) was not significant.

As seen in Figure 2, above the line of discrepancy (x = –y), the response surface rises from the bottom left corner (−2, 2) toward the top right corner of the graph (2, −2) where GRMF (X) is highest and GRMS (Y) is lowest, and IR (Z) is high. In support of hypothesis 2, the increase in IRAAS as this discrepancy increased was significant (Table 2), as reflected in the significant positive slope for the response surface above the line of discrepancy (x = –y) = 0.60, *SE* = 0.22, *t* = 2.754, *p* = 0.006. The effect size was small to medium, Cohen's *d* = 0.31. The curvature above the line of discrepancy (x = y) was not significant.

### GRMF GRMS congruence and discrepancy predicting PHQ-9 across low, mean, high IRAAS levels

**Figure 3:**
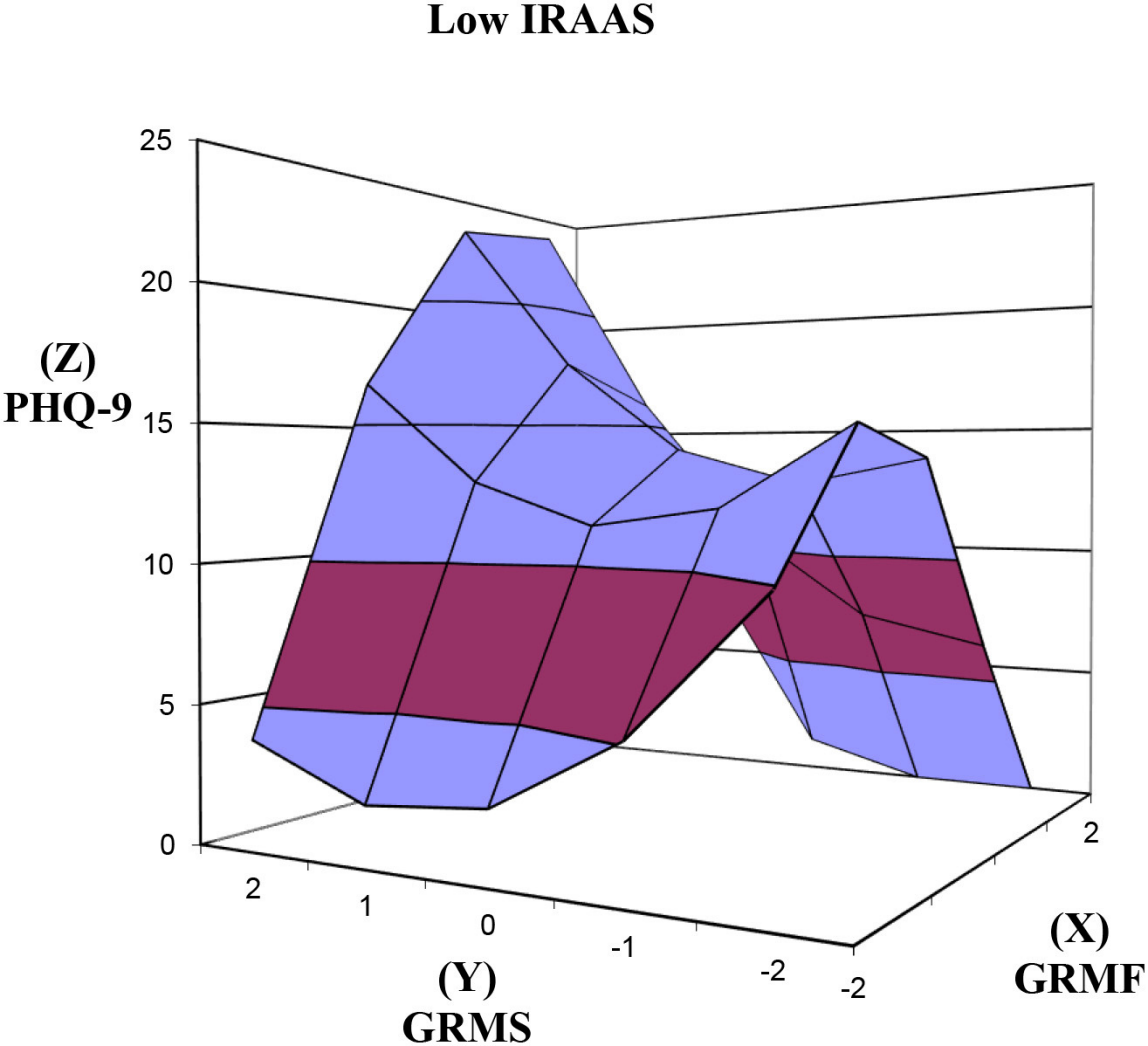
Response surfaces predicting PHQ-9 as a function of congruence and discrepancy between GRMF and GRMS among Asian American women with low level of internalized racism. PHQ-9, Patient Health Questionnaire-9; IRAAS, Internalized Racism in Asian American Scale; GRMF, Gendered Racial Microaggressions Frequency; GRMS, Gendered Racial Microaggressions Stress; Line of congruence (X = Y; back to front); Line of discrepancy (X = –Y; left to right); point of lowest congruence (−2, −2; front corner); point of highest congruence (2, 2; back corner); congruence increases from front to back corner; right most corner (2, −2) = discrepancy between high GRMF-low GRMS; left most corner (−2, 2) = discrepancy between low GRMF and high GRMS.

At low IRAAS (Figure 3), in support of hypothesis 3, the increase in PHQ-9 as GRMF GRMS congruence increases was significant (Table 2) as reflected in the significant positive slope for the response surface above the line of congruence (x = y) = 2.01, *SE* = 0.84, *t* = 2.394, *p* = 0.021. The effect size was small, Cohen's *d* = 0.27. Contrary to hypothesis 3, the slope above the line of discrepancy (x = –y) was not significant in predicting PHQ-9. The congruence and discrepancy curvatures were not significant.

**Figure 4:**
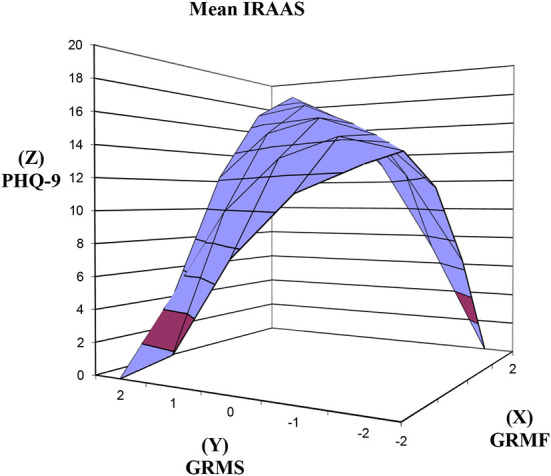
Response surfaces predicting PHQ-9 as a function of congruence and discrepancy between GRMF and GRMS among Asian American women with mean level of internalized racism. PHQ-9, Patient Health Questionnaire-9; IRAAS, Internalized Racism in Asian American Scale; GRMF, Gendered Racial Microaggressions Frequency; GRMS, Gendered Racial Microaggressions Stress; Line of congruence (X = Y; back to front); Line of discrepancy (X = –Y; left to right); point of lowest congruence (−2, −2; front corner); point of highest congruence (2, 2; back corner); congruence increases from front to back corner; right most corner (2, −2) = discrepancy between high GRMF-low GRMS; left most corner (−2, 2) = discrepancy between low GRMF and high GRMS.

At mean IRAAS (Figure 4), in support of hypothesis 3, the increase in PHQ-9 as GRMF GRMS congruence increases was significant (Table 2) as reflected in the significant positive slope for the response surface above the line of congruence (x = y) = 1.63, *SE* = 0.42, *t* = 3.890, *p* < 0.001. The effect size was small to medium, Cohen's *d* = 0.44. Contrary to hypothesis 3, the slope above the line of discrepancy (x = –y) was not significant in predicting PHQ-9. The congruence and discrepancy curvatures were not significant.

**Figure 5:**
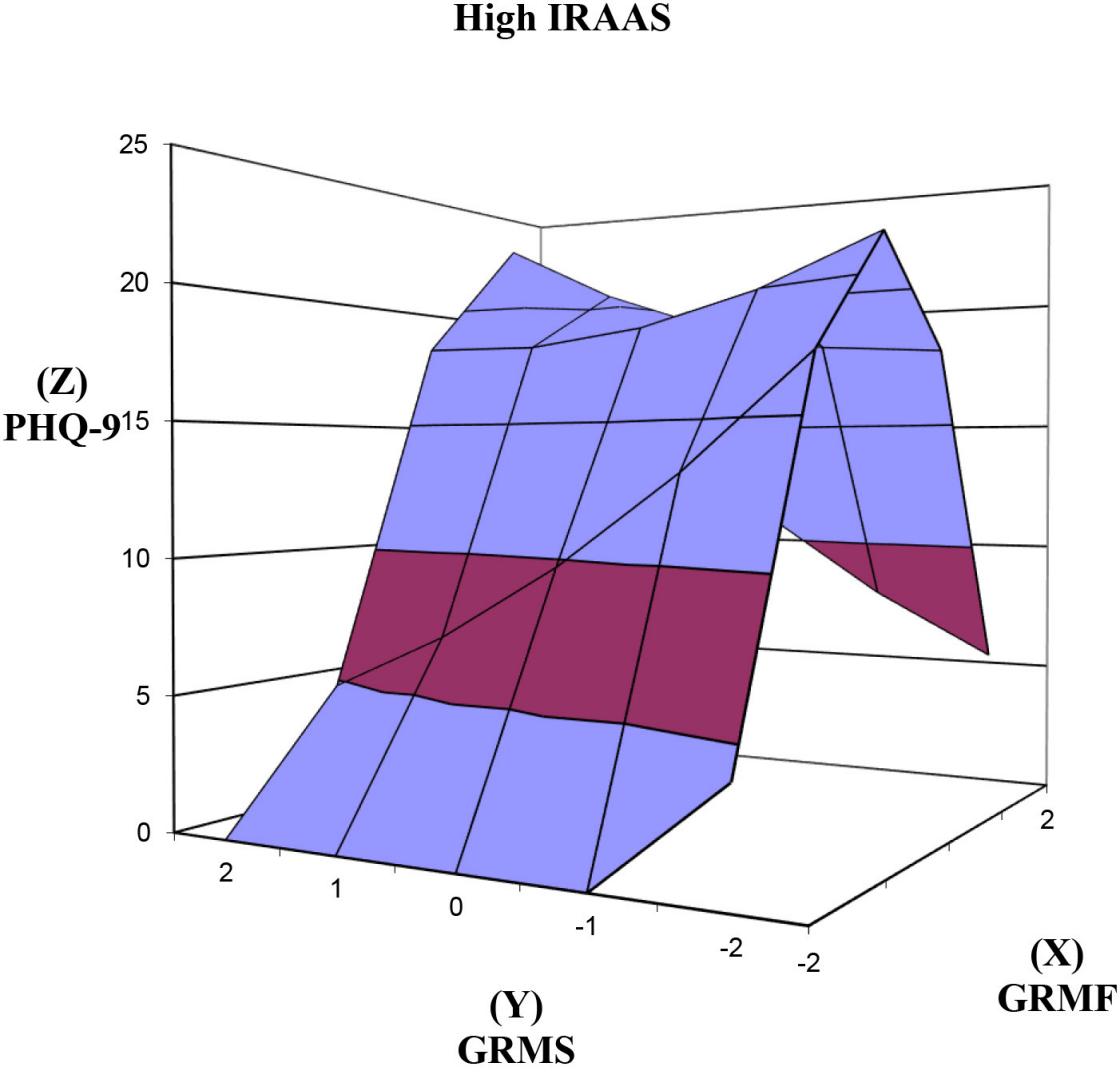
Response surfaces predicting PHQ-9 as a function of congruence and discrepancy between GRMF and GRMS among Asian American women with high level of internalized racism. PHQ-9, Patient Health Questionnaire-9; IRAAS, Internalized Racism in Asian American Scale; GRMF, Gendered Racial Microaggressions Frequency; GRMS, Gendered Racial Microaggressions Stress; Line of congruence (X = Y; back to front); Line of discrepancy (X = –Y; left to right); point of lowest congruence (−2, −2; front corner); point of highest congruence (2, 2; back corner); congruence increases from front to back corner; right most corner (2, −2) = discrepancy between high GRMF-low GRMS; left most corner (−2, 2) = discrepancy between low GRMF and high GRMS.

Finally, as hypothesized, the slopes above the line of congruence (x = y) and the line of discrepancy were not significant at high IRAAS (Figure 5). The congruence and discrepancy curvatures were not significant.

## Discussion

We used polynomial response surface analysis to examine congruence and discrepancy between frequency, or perceived number of GRM encounters and stress appraisal of GRM experienced among AAW. We found that greater congruence between the amount of GRM encountered and the amount of stress experienced significantly predicted depressive symptoms. Thus, as expected, AAW who encounter more GRM and a corresponding amount of more stress reported significantly more depressive symptoms than those encountering less GRM and lower amount of stress. The congruence between frequency and stress appraisal of GRM also had a significant relationship with internalized racism but at small effect. In terms of discrepancy, we found that higher frequency-lower stress discrepancy was significantly related to greater internalized racism, suggesting that AAW who experience more GRM but feel little stress are likely to hold greater beliefs of internalized racism. When we examined the congruence and discrepancy and their relationships with depressive symptoms across levels of internalized racism (low, mean, high), greater congruence was significantly associated with greater depressive symptoms at low to mean internalized racism levels but not at a high level. These findings suggest that the congruence and discrepancy between GRM encounters and the appraisal of the resulting stress carry important nuances to understanding how GRM affects the mental health of AAW.

Prior research informed by the stress process model has noted the importance of assessing encounters of racial discrimination, with the understanding that greater exposure can adversely impact health as the perceived stressors from discrimination exceeds available coping resources (14–16). While commonly used measures such as the Everyday Discrimination Scale (EDS) help to capture the frequency of discrimination, and the General Ethnic Discrimination Scale (GED) assesses the stressfulness of discrimination, studies have yet to demonstrate the concurrent interplay between dimensions of frequency and stress appraisal (16, 18). Thus, the current study sought to examine whether the degree of congruence and discrepancy between the frequency and stress appraisal of GRM would be associated with AAW depressive symptoms. In partial support of our first hypothesis, findings indicate that AAW who report proportional amounts of stress from GRM encounters (i.e., congruence) are more likely to experience an increase in their depressive symptoms.

These results align with previous research that highlights the role of acculturation, particularly among Asian American individuals raised in the U.S. who are more apt to identify and understand the negative implications of discrimination, and report more depressive symptoms as a result (25, 26). Further, while some research has pointed to ethnic identity (i.e., a sense of clarity and pride in one's ethnic group) as an important buffer against discrimination, there is also evidence to suggest that high ethnic identity among Asian Americans may exacerbate the link between discrimination and depressive symptoms, as experiences of chronic discrimination may also increase sensitivity to rejection or devaluation of one's race/ethnicity (42–44). Another possible explanation may be related to the contexts where AAW encounter GRM, such as the workplace, where it is difficult to walk away from GRM perpetuated by supervisors or colleagues that AAW must interact with daily, and can serve as an added stressor on top of existing job demands and responsibilities (45, 46). Given the high rates of suicide among AAW in their late adolescence and emerging adulthood, future research should assess both frequency and stress appraisal to understand what factors drive proportional amounts of stress in relation to GRM encounters, and thereby increase their risk for depressive symptoms.

Previous studies have noted the importance of examining the role of internalized racism among Asian Americans, particularly as it may lead to the belief in harmful racist stereotypes and acceptance of one's racial inferiority that can lead to worse mental health (9, 35). Yet, few studies have examined the role of internalized racism among Asian American women that encounter GRM (7). Hence, the current study sought to assess whether AAW who report greater congruence between GRM frequency and appraised stress are more likely to experience higher internalized racism. In support of our second hypothesis, our findings indicate AAW who report proportional amounts of stress from their encounters with GRM are more likely to experience significant increases in internalized racism. This finding is consistent with Choi et al. (32) conceptualization of internalized racial oppression among Asian Americans, and suggests that AAW that endorse oppressive ideologies of GRM (e.g., AAW as submissive and exoticized) may feel more self-negativity and an overall devaluation of their identity as an Asian American woman. However, given the small effect size, these results should be interpreted with caution, as the internalized racial oppression dimensions of weakness stereotype and appearance bias can lead to different gendered experiences for AAW as compared to Asian American men, and may instead manifest as more of a complex self-protective strategy for AAW (34).

Indeed, our findings suggest AAW may engage with internalized racism as a self-protective strategy against poor mental health. The results indicate a discrepancy of high frequency but low stress was significant in predicting internalized racism among AAW, suggesting that AAW that perceive more GRM, yet feel little stress as a result, may hold greater beliefs of internalized racism. In particular, when viewed through the lens of appropriated racial oppression, AAW may choose to adapt to oppressive ideologies, instead of passively accepting the negative messaging associated with GRM (10, 33). For instance, AAW may consciously or subconsciously endorse assumptions of their submissiveness and fetishized exoticization, and find ways to downplay, deny, and even justify their experiences of GRM (33). Accordingly, weakness stereotypes and appearance bias may operate in intricate ways, such that being meek is considered desirable among Asian women (i.e., aligns with the dominant White supremacist society's views of AAW as submissive). Thus, AAW that report high levels of internalized racial oppression, may not view GRM as discriminatory, and therefore not perceive these events as stressful, contributing to a greater discrepancy between the perceived number of GRM (i.e., frequency) and stress appraisal. However, it is important to note that not all self-protective strategies will produce less stress. In fact, a recent phenomenological study investigating how Black female school leaders cope with gendered racism, finds that across a range of coping mechanisms, including self-protective strategies, there is a cost to coping, where the constant struggle to maintain their emotional health takes a toll on their mental and physical health (47). More research is needed to understand how AAW cope with GRM, and how different coping strategies are linked with stress.

Further subtleties in AAW's mental health emerged when examining the congruence between frequency and stress and their relationship with depressive symptoms across levels of internalized racism. At low and mean levels of internalized racism, greater congruence between frequency and stress was significantly associated with greater depressive symptoms, but not at high levels of internalized racism. Given the small effects size found for AAW at low levels of internalized racism, depressive symptoms that AAW experienced may be attributed more to the stress they experience from GRM encounters. However, mean levels of internalized racism appear to confer the greatest risk, at medium effect size, which suggests that AAW that report a proportional amount of stress from their encounters with GRM may feel more self-hate as they internalize negative messaging from GRM, thereby increasing their risk for depressive symptoms.

Conversely, at high levels of internalized racism, AAW reported no significant risk for depressive symptoms. A possible explanation for the lack of significance, is that AAW may adopt a more self-protective survival strategy to avoid feelings of self-hate and self-negativity, and instead find ways to identify with and utilize aspects of GRM that can help diminish their depressive symptoms. For example, some AAW may endorse views of Asian American women as hypersexualized and exoticized, such that being treated as a sexual object or as submissive may not be perceived as a stressful discriminatory event, particularly if they themselves choose to “play the part.” Despite results indicating that AAW at high levels of internalized racism do not report depressive symptoms, which could be interpreted as having potential protective benefits, it is important to note that AAW may still incur self-deprecating costs to their mental health in the long-term for becoming “honorary Whites” or “White adjacent” and upholding detrimental racist ideologies perpetuated through White norms and beliefs (48). In addition, research on the John Henryism hypothesis among African American women suggests that AAW that perform as the exotic and hypersexualized Asian women may be engaging in a form of high effort coping in response to prolonged exposures to GRM, and will likely experience significant costs to their physical and mental health in the long term (49). Accordingly, future research would need to assess other psychological factors such as color-blind racial attitudes and the role of AAW's gender racial identity to understand how AAW's level of critical awareness of their own gender racial identity and gendered racism in the U.S. can affect their perceptions and stress appraisal of GRM, thereby shaping important nuances in AAW's mental health, particularly when considering the role of internalized racism.

### Limitations and directions for research

Despite the novel contributions of our study, there are several limitations that inform future research. First, the data was cross-sectional and we are limited to interpreting how the levels of frequency and stress appraisals are associated with concurrent levels of depressive symptoms reported by the participants. As we conceptualized, we assume that when AAW experience GRM, they subsequently feel a certain level of stress based on their appraisal, which may then contribute to the development of depressive symptomatology. This sequence would need to be examined by replicating our study using longitudinal data. Second, although our sample represents diverse Asian ethnicities, the majority of the participants are highly educated young adult individuals with East Asian roots which limit our generalizability. Our findings would need to be extended with larger samples of South Asian American and Southeast Asian American individuals and across varying educational status and age groups. Third, the GRMS and IRAAS scales have been developed with greater samples of East Asian American participants and conceptualization that may align more with the stereotypes of East Asian Americans. While there are shared components of the GRM and internalized racism across ethnicities, it would be important for future studies to explicate the unique differences in these experiences (e.g., colorism) for greater culture-specific understanding. Fourth, the measures used in the study were self-report and may be prone to self-report bias. In particular, self-report appraisal of stress is subjective and does not reflect objective indications of actual stress levels that could be obtained *via* physiological measures (e.g., cortisol swab). Furthermore, while we examined internalized racism as one factor that could differentiate the recognition and stress appraisal process of GRM events, there may be additional factors that could help contextualize the variations such as acculturation. For example, Asian Americans who are less acculturated have been found to report greater color-blind racial attitudes compared to more acculturated individuals (29). Futures studies can expand our findings by using more objective markers of stress appraised from GRM events. Of note, qualitative studies can help explore additional contextual factors such as acculturation and color-blind racial attitudes to understand the complexity of AAW's GRM experiences.

### Implications for practice and advocacy

Our findings provide implications for interventions geared toward mitigating the harmful effects of GRM among AAW. Given that depressive symptoms are significantly associated with those who encounter a high level of GRM and appraise a high level of stress, it may be useful to develop interventions that help to externalize the harmful effects of GRM and lessen the stressfulness. For instance, Miller et al. (50) suggest the importance of externalizing the harmful internalizations stemming from racism encounters so that it does not lead to the development of psychopathology. Externalization may be achieved by helping AAW engage at the individual and community levels with counter-narratives that signify the positive self-concepts and pride in being an Asian American woman in their respective ethnic cultures and create social support and community around the shared experiences of GRM. For instance, interventions such as the Asian Women's Action for Resilience and Empowerment [AWARE; (51)] that focuses on gender- and culture-specific group psychotherapy intervention for AAW dealing with interpersonal violence and trauma could be tailored to help AAW experiencing depressive symptoms from GRM.

Individual and community level interventions can also target internalized racism, which has been found to dictate the recognition and stress appraisal of GRM among AAW. In particular, those who harbor high levels of internalized racism may be instilling beliefs that reinforces Asian inferiority and disavowal of the reality of White supremacist oppression. This may be a complex state of survival by succumbing to the dominant group and the oppressive narrative but one that can lead to self-destructive implications given the self-negativity and -erasure [e.g., suicide ideation; (7)]. Conversations and advocacy around this issue must occur in tandem with a critical evaluation of assimilationist and internalized Whiteness ideals in Asian American communities, as well as the White dominant society that continues to reinforce systems of oppression against AAW (52).

## Data availability statement

The raw data supporting the conclusions of this article will be made available by the authors, without undue reservation.

## Ethics statement

The studies involving human participants were reviewed and approved by University of Maryland-College Park Institutional Review Board. The patients/participants provided their written informed consent to participate in this study.

## Author contributions

BK is the principal investigator who led the conceptualization, data collection, methodology, data analysis, and manuscript writing. MW contributed to manuscript writing and editing. All authors contributed to the article and approved the submitted version.

## Conflict of interest

The authors declare that the research was conducted in the absence of any commercial or financial relationships that could be construed as a potential conflict of interest.

## Publisher's note

All claims expressed in this article are solely those of the authors and do not necessarily represent those of their affiliated organizations, or those of the publisher, the editors and the reviewers. Any product that may be evaluated in this article, or claim that may be made by its manufacturer, is not guaranteed or endorsed by the publisher.
